# Epidemiology of Spinal Cord Injuries and Risk Factors for Complete Injuries in Guangdong, China: A Retrospective Study

**DOI:** 10.1371/journal.pone.0084733

**Published:** 2014-01-28

**Authors:** Rui Yang, Lan Guo, Peng Wang, Lin Huang, Yong Tang, Wenhao Wang, Keng Chen, Jichao Ye, Ciyong Lu, Yanfeng Wu, Huiyong Shen

**Affiliations:** 1 Department of Orthopedics, Sun Yat-sen Memorial Hospital, Sun Yat-sen University, Guangzhou, People's Republic of China; 2 Biotherapy Centre, Sun Yat-sen Memorial Hospital, Sun Yat-sen University, Guangzhou, People's Republic of China; 3 Department of Medical Statistics and Epidemiology, School of Public Health, Sun Yat-sen University, Guangzhou, People's Republic of China; St Michael's Hospital, University of Toronto, Canada

## Abstract

**Background:**

Spinal cord injuries are highly disabling and deadly injuries. Currently, few studies focus on non-traumatic spinal cord injuries, and there is little information regarding the risk factors for complete injuries. This study aims to describe the demographics and the injury characteristics for both traumatic and non-traumatic spinal cord injuries and to explore the risk factors for complete spinal cord injuries.

**Methods:**

A retrospective study was performed by reviewing the medical records of 3,832 patients with spinal cord injuries who were first admitted to the sampled hospitals in Guangdong, China. The demographics and injury characteristics of the patients were described and compared between the different groups using the chi-square test. Logistic regression was conducted to analyze the risk factors for complete spinal cord injuries.

**Results:**

The proportion of patients increased from 7.0% to 14.0% from 2003 to 2011. The male-to-female ratio was 3.0∶1. The major cause of spinal cord injuries was traffic accidents (21.7%). Many of the injured were workers (36.2%), peasants (22.8%), and unemployed people (13.9%); these occupations accounted for 72.9% of the total sample. A multivariate logistic regression model revealed that the OR (95% CI) for male gender compared to female gender was 1.25 (1.07–1.89), the OR (95%CI) for having a spinal fracture was 1.56 (1.35–2.60), the OR (95%CI) for having a thoracic injury was 1.23 (1.10–2.00), and the OR (95%CI) for having complications was 2.47 (1.96–3.13).

**Conclusion:**

The proportion of males was higher than the proportion of females. Workers, peasants and the unemployed comprised the high-risk occupational categories. Male gender, having a spinal fracture, having a thoracic injury, and having complications were the major risk factors for a complete injury. We recommend that preventive measures should focus on high-risk populations, such as young males.

## Introduction

In a spinal cord injury (SCI), the structures and functions of the spinal cord are damaged by trauma, inflammation, tumors or other causes, resulting in dysfunction of motion, feeling, sphincters and autonomic nerves below the damaged plane. A SCI is a highly disabling and deadly injury. Based on their etiology, SCIs can be divided into two different groups: traumatic spinal cord injuries (TSCI) and non-traumatic spinal cord injuries (NTSCI) [1]. The incidence and prevalence of spinal injuries have been increasing, with the incidence rate estimated at 15 to 40 cases per million worldwide, although injury prevention initiatives have tried to reduce the occurrence of SCIs [2]. With the modernization of society, SCI incidence increases year after year. Spinal cord injuries are highly disabling and concentrated in young adults, so they bring great pain to the affected individuals and their families. In addition to the substantial burden for the affected individuals and their families, society must bear the cost of healthcare treatments, rehabilitation and lost productivity[3]. The epidemiological characteristics of SCIs obviously vary in different countries, in regions with different economic levels or in different economic periods. The mean age of the SCI patient in developed countries is higher than in developing countries over the same time period; the reason may be related to the aging of the populations in developed countries and/or to the larger male-to-female ratio in developing countries in relation to developed countries [4]. France reported an SCI incidence rate of 19.4 per million, or an average of 934 new cases each year [5]. Finland reported an annual SCI incidence of 28 per million in 2005 [6]. Canada reported that the incidence of TSCIs in people aged 15 to 64 was 42.4 per million, and for people over the age of 65, the incidence was 51.4 per million between January 1997 and June 2001 [7]. Australia reported that the average estimated incidence rates of traumatic and non-traumatic spinal cord injuries were 3.8 and 6.5 per million children younger than 15 years of age, respectively [8]. The United States reported an annual average SCI incidence of 40 per million in 2012 [9]. Taiwan reported that the incidence rate of pediatric spinal cord injuries is 5.99 per million person-years [10]. Young adults are the age group at the highest risk of SCIs: the 21-to-30 age group has the highest number of patients, and the number of SCIs is greater in males than in females. This finding is possibly related the fact that more young men are engaged in dangerous outdoor activities, while most women work in the household or perform other relatively less dangerous work.

According to the international standards set forth by the American Spinal Injury Association (ASIA), the severity of an injury is categorized as either complete or incomplete. A complete injury is defined as the absence of sensory and motor function in the lowest sacral segments [11]. The proportion of SCIs that were complete was 67% over the past 30 years; this value has decreased to 45% over the past decade in Beijing [12]. This decrease may be due to improvements in first aid technology that allow SCI patients to receive timely and appropriate treatment. Furthermore, these improvements may effectively avoid the aggravation of the injury and delays in treatment. Canada reported that complete injuries accounted for 35% of the SCIs in 2006. The incidence of complete injury was higher in the thoracic segments than in the lumbar and cervical segments [13]. Tianjin reported that 100% of thoracic SCIs resulted in complete injuries, whereas 46.7% and 60% of cervical and lumbar SCIs, respectively, presented as incomplete injuries. SCI is often associated with fractures at other locales and brain injuries [14]. Understanding the severity of these injuries has guided the treatment options for SCI patients. SCI patients are at high risk of medical complications that can prolong their hospitalization, affect their treatment and impair their recovery. The complications for SCI patients include fever, pulmonary complications, electrolyte disturbances, spasms, pain, urinary tract infections, autonomic dysreflexia, cardiovascular disease, osteoporosis and fractures, myositis ossificans, deep vein thromboses, bedsores, and pruritus. A group of Italian investigators reported that the most common complication among SCI patients was urinary tract infection, followed by pain and spasms [15]. In addition, an Italian study reported that patients with multiple injuries, such as associated brain injuries, are affected by more severe neurological lesions [16]. To date, there has been no obvious breakthrough in the clinical treatment of SCI; therefore, the emphasis has been on prevention. As stated above, economic and social structures, politics and cultural traditions play important roles in the differences in the epidemiological characteristics of SCI patients in different regions. This retrospective study focuses on the demographics and epidemiological characteristics of SCI patients in Guangdong, China. The goals of the study were to explore effective prevention and control measures so that health-related institutions can develop policies to make the best use of medical resources.

## Methods

### Study subjects

In China, hospitals are categorized by medical level into three grades (third-grade is the best), and each grade is divided into three classes from better to worse: A, B, and C. The study used typical sampling techniques to select partial second-grade class-A hospitals (mainly capturing general city and county hospitals and some large-scale affiliated hospitals) in Guangzhou, Shantou, Shaoguan, and Zhanjiang according to the geography and the economic characteristics of Guangdong province. The subjects were 3,832 patients with spinal cord injuries who were first admitted to the sampled hospitals between 2003 and 2011. For this process, spinal cord injury was defined using the international definition as the occurrence of an acute lesion on the neural elements in the spinal canal (spinal cord and cauda equina), resulting in temporary or permanent sensory deficits, motor deficits, or bladder/bowel dysfunction.

### Ethical statement

This study was approved by the ethics committee of the Sun Yat-sen Memorial Hospital of Sun Yat-sen University. The research assistants collected all the required data by reviewing the medical records of the sampled hospitals, concealed any identifiable details about the patients, and recorded these blind data in a privacy-enhanced database. Next, the researchers were allowed to read and analyze the data. The institutional review boards of the sampled hospitals permitted the review process, and the need for the patients' written informed consent was waived.

### Study methods

This retrospective study used questionnaires to review medical records and to obtain the relevant data. The questionnaire about the patients included the patients' age, gender, residence, ethic group, occupation, marital status, time of injury, hospital admission and discharge date, cause of injury, level of injury, severity of injury, acceptance of surgical treatment, traction, hyperbaric oxygen therapy, rehabilitation therapy and so on. The time period was subdivided into three sub-periods: 2003–2005, 2006–2008, and 2009–2011. The patients were divided into four age groups: ≤20, 21–40, 41–60, and ≥61 years. The place of residence was defined as either Guangdong or other, and the patient's ethnic group was classified as either Han or other. Occupations were categorized as peasants, workers, students, retired individuals, unemployed individuals, self-employed individuals, enterprise staffs, civil servants and all other occupations not classified above. Marital status was defined as married, unmarried, divorced, or widowed. The specific causes of injury were classified as non-traumatic causes or traumatic causes, which included traffic accidents, being struck by falling objects, high falls (height>1 m), low falls (height≤1 m), crushing injuries, violence, and sports-related injuries. The levels of injury were associated with the mechanism of the injury and the structures of the spinal column: they were separated into cervical, thoracic, and lumbar spine segments. For the purposes and the practical conditions of the present study, the severity of the injury causing the neurological deficits was categorized as either complete or incomplete according to the international standards set forth by the American Spinal Association (ASIA). A complete injury was defined as the absence of sensory and motor function in the lowest sacral segments. An incomplete injury was defined as an SCI in which sensory and/or motor function (≥3 segments) is partially preserved below the level of injury and including the lowest sacral segments [17].

### Statistical analysis

All the data were entered twice by two investigators independently using Epidata 3.1 and analyzed using IBM SPSS Statistics 20.0 (IBM, Armonk, NY, USA). In the study, a descriptive analysis was performed on the demographics and injury characteristics to help with the prevention of SCI. Categorical and continuous data are reported as proportions and means ± SDs. Chi-square tests were used to test the differences between the categorical variables. A logistic regression model was used to analyze the relationships between two or more variables so that a dependent qualitative variable could be predicted by the other(s). The odds ratios derived from the logistic regression analysis estimated the likelihood of a given outcome among persons with a particular characteristic relative to those without that characteristic. In this study, the outcome of interest was the severity of the injury. Unconditional univariate logistic regression models were used to estimate the odds ratios (ORs) and 95% confidence intervals (CIs) for the associations between the severity of the injury and the injury/demographic characteristics. The multivariate logistic regression models included the significant variables from the univariate logistic regression analysis to determine the risk factors for complete injuries based on the ORs and 95% CIs. An OR>1 with *P*<0.05 indicated a risk factor and vice versa. The variables used the Forward: LR method in the equation. All the statistical tests were two-sided, and a *P* value less than 0.05 was considered significant.

## Results

**Figure 1:**
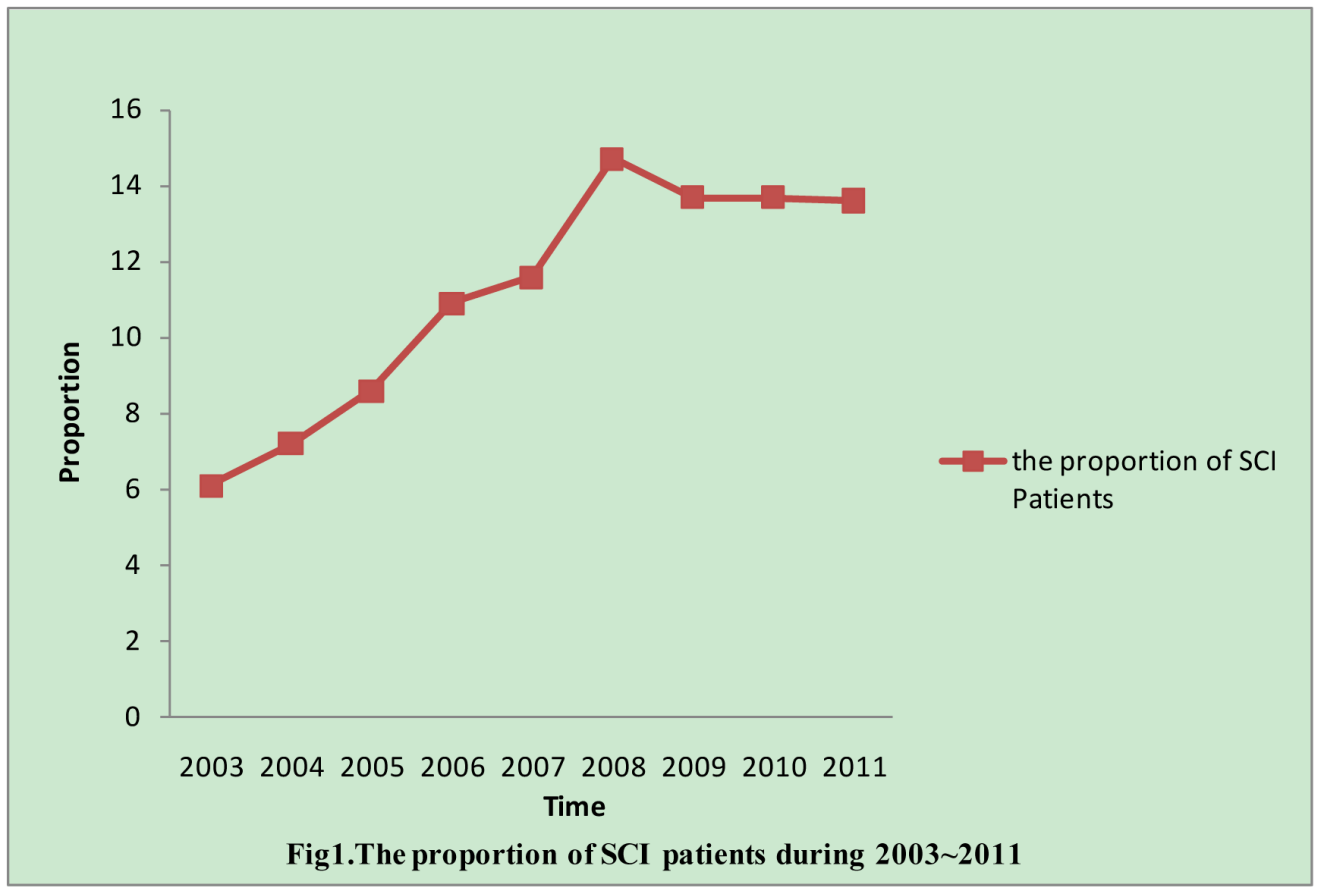
The proportion was calculated by dividing the number of SCI patients each year by the total number n (n = 3832).

The study included the medical records of 3,832 patients with spinal cord injuries from 2003 to 2011. Although the total number of ward beds in the study hospitals remained unchanged over the study period, the proportion of SCI admissions increased (**Fig. 1**). Because the study was not population-based, the results can only be presented as proportions and percentages and not as rates.

### General demographics

Of the 3,832 individuals with SCIs, 2,892 were male (75.5%), 940 were female (24.5%), and the male-to-female ratio was approximately 3.0∶1. The patients' ages ranged from 1 to 94 years, the mean age was 42.4±15.3 years, and the median age was 42.0 years old. The age group with the greatest percentage of patients was 41–60 years (36.4%), followed by 21–40 years (35.5%); thus, young adults are the high-risk population. The occupations of the patients with SCIs included workers (36.2%), peasants (22.8%), unemployed persons (13.9%), retired persons (6.9%), civil servants (3.4%), students (3.0%), self-employed persons (2.1%), enterprise staffs (1.1%), and other occupations that were difficult to classify (10.5%). The most common occupational groups were workers (36.2%), peasants (22.8%), and unemployed persons (13.9%), who together accounted for 72.9% of the total. The main ethnic group was Han, which accounted for 92.7% of the SCI patients, and the other ethnic groups included Zhuang (3.2%), Miao (3.1%) and Tujia (1.0%). The proportion of patients who resided in Guangdong was 69.3%. More than 70% of the SCI patients were married (**Table 1**).

**Table 1:** Demographic characteristics of patients with SCI.

Variables	Numbers (%)
**Total**	3832 (100.0)
**Gender**	
Male	2892 (75.5)
Female	940 (24.5)
**Age** a **(years)**	
≤20	376(9.8)
21–40	1361 (35.5)
41–60	1394 (36.4)
≥61	478 (12.5)
**Occupation**	
Workers	1387 (36.2)
Peasants	873 (22.8)
Unemployed	533 (13.9)
Retired	265 (6.9)
Other occupations^b^	371 (9.6)
Other^c^	403 (10.5)
**Ethnic group**	
Han	3554 (92.7)
Zhuang	122 (3.2)
Miao	119 (3.1)
Tujia	37 (1.0)
**Residence**	
Guangdong	2654 (69.3)
Other	919 (24.0)
Unknown	259 (6.7)
**Marital status**	
Married	2754 (71.9)
Unmarried	776 (20.3)
Divorced	12 (0.3)
Widowed	8 (0.2)
Unknown	282 (7.3)

a A total of 223 patients' medical records were missing age information.

b Other occupations included self-employed people, civil servants, students, and enterprise staff.

c Other referred to all other occupations not classified above.

### Etiology of the injuries

Spinal cord injuries can be divided into traumatic and non-traumatic injuries. The four main causes of injury were traumatic injuries, including traffic accidents (21.7%), being struck by falling objects (19.5%), crushing injuries (15.1%) and high falls (9.8%), followed by low falls (3.8%), violence (3.2%), non-traumatic causes (8.2%), sports-related injuries (0.7%), and other unknown causes (18.1%). In this study, inflammation (0.9%), tumors (0.9%), ossification (2.9%), degenerative damage (2.8%) and vascular damage (0.7%) comprised the non-traumatic causes. Over the study period, the number of patients admitted to the sampled hospitals increased steadily. The final data revealed that traffic accidents were the leading cause of SCIs in all three study periods, but there were statistically significant differences in the distribution of the etiology during the different periods (*P*<0.05) (**Table 2**). The four main causes of injury were traffic accidents, high falls, being struck by falling objects, and crushing injuries for both males and females. However, the leading cause for male patients was traffic accidents, whereas high falls were the main cause of injury for female patients. There were significant differences in the gender distribution by etiology (*P*<0.05) (**Table 2**). The age distribution by etiology is presented. The rates of traffic accidents, high falls, and being struck by falling objects were high among the patients between the ages of 21 and 60 years. Among those older than 60 years, low falls were the most common cause of SCIs. Statistically significant differences were noted among the age groups with regard to etiology (*P*<0.001) (**Table 2**). As shown in **Table 3**, traffic accidents were the leading cause of injury for workers, peasants and unemployed people, accounting for 22.0%, 23.4% and 25.9% of the injuries, respectively. Being struck by falling objects was the second most common cause of injury for both workers and the unemployed, while the second most common cause for peasants was high falls. The differences in the etiologies between occupations were statistically significant (*P*<0.001). Traffic accidents were the leading cause of injury for married patients and accounted for 22.5% of the injuries, but there were not significant differences in the distribution of marital status by etiology (*P*>0.05) (**Table 3**).

**Table 2:** Distribution of SCI patients' characteristics by etiology.

Variables	Etiologies, n (%)								*P-value*
	Traffic accidents	Struck^a^	Crushing injuries	High falls	Other^b^	Non-traumatic^c^	Unknown	Total	
**Total**	831 (21.7)	746 (19.5)	577 (15.1)	374 (9.8)	298 (7.7)	313 (8.2)	692 (18.1)	3832 (100.0)	
**Period**									0.023
2003∼2005	235 (22.1)	189 (17.8)	175 (16.5)	110 (10.4)	78 (7.3)	88 (8.3)	187 (17.6)	1062 (27.7)	
2006∼2008	291 (22.3)	254 (19.5)	193 (14.8)	122 (9.4)	102 (7.8)	122 (9.4)	220 (16.9)	1304 (34.0)	
2009∼2011	305 (20.8)	303 (20.7)	210 (14.3)	142 (9.7)	118 (8.0)	103 (7.0)	285 (19.4)	1466 (38.2)	
**Gender**									0.036
Male	713 (24.7)	620 (21.4)	469 (16.2)	240 (8.3)	265 (9.2)	245 (8.5)	340 (11.8)	2892 (75.4)	
Female	118 (12.6)	126 (13.4)	109 (11.6)	134 (14.3)	33 (3.5)	68 (7.2)	352 (37.4)	940 (24.6)	
**Age** d **(y)**									<.001
≤20	100 (26.6)	41 (10.9)	50 (13.3)	52 (13.8)	35 (9.3)	25 (6.6)	73 (19.4)	376 (9.8)	
21–40	365 (26.8)	280 (20.6)	208 (15.3)	104 (7.6)	121 (8.9)	39 (2.9)	244 (17.9)	1361 (35.5)	
41–60	346 (24.8)	318 (22.8)	207 (14.8)	136 (9.8)	128 (9.2)	68 (4.9)	191 (13.7)	1394 (36.4)	
≥61	13 (2.7)	96 (20.0)	90 (18.8)	73 (15.3)	81 (16.9)	8 (1.7)	117 (24.5)	478 (12.5)	

NOTE. Total number of cases: N = 3832.

a Struck = struck by falling objects.

b Other included low falls, violence, and sports-related injuries.

c Non-traumatic referred to inflammation, tumor, ossification, degenerative damage, and vascular damage.

d A total of 223 patients' medical records were missing age information.

**Table 3:** Distribution of SCI patients' characteristics by etiology (continued).

Variables	Etiologies, n (%)								*P-value*
	Traffic accidents	Struck^a^	Crushing injuries	High falls	Other^b^	Non-traumatic^c^	Unknown	Total	
**Occupation**									<.001
Workers	305 (22.0)	257 (18.5)	245 (17.7)	103 (7.4)	102 (7.4)	139 (10.0)	236 (17.0)	1387 (36.2)	
Peasants	204 (23.4)	104 (11.9)	96 (11.0)	162 (18.6)	93 (10.7)	30 (3.4)	184 (21.1)	873 (22.8)	
Unemployed	138 (25.9)	132 (24.8)	88 (16.5)	23 (4.3)	15 (2.8)	33 (6.2)	104 (19.5)	533 (13.9)	
Retired	88 (33.2)	13 (4.9)	24 (9.0)	56 (21.1)	28 (10.6)	45 (17.0)	11 (4.2)	265 (6.9)	
Other occupations^d^	54 (14.6)	121 (32.6)	85 (22.9)	22 (5.9)	32 (8.6)	28 (7.5)	29 (7.8)	371 (9.7)	
Other	42 (10.4)	119 (29.5)	40 (9.9)	8 (2.0)	28 (6.9)	38 (9.4)	128 (31.8)	403 (10.5)	
**Marital status**									0.810
Married	619 (22.5)	542 (19.7)	389 (14.1)	225 (8.2)	209 (7.6)	164 (6.0)	606 (22.0)	2754 (71.9)	
Unmarried	156 (20.1)	174 (22.4)	165 (21.3)	118 (15.2)	35 (4.5)	72 (9.3)	56 (7.2)	776 (20.3)	
Divorced	3 (25.0)	5 (41.7)	0 (0)	0 (0)	2 (16.7)	0 (0)	2 (16.7)	12 (0.3)	
Widowed	2 (25.0)	1 (12.5)	0 (0)	0 (0)	2 (25.0)	2 (25.0)	1 (12.5)	8 (0.2)	
Unknown	51 (18.1)	24 (8.5)	24 (8.5)	31 (11.0)	50 (17.7)	75 (26.6)	27 (9.6)	282 (7.3)	

NOTE. Total number of cases: N = 3832.

a Struck = struck by falling objects.

b Other included low falls, violence, and sports-related injuries.

c Non-traumatic referred to inflammation, tumor, ossification, degenerative damage, and vascular damage.

d Other occupations included self-employed people, civil servants, students, and enterprise staff.

### Level of injury

As shown in **Table 4**, the level of injury at admission for the patients with SCIs, as a group, ranged from cervical to lumbar spine. The highest number of individuals who suffered a SCI suffered a cervical injury (1,720 cases), followed by thoracic and lumbar injuries (1,264 and 941 cases, respectively). The sum of the injuries at all three levels was higher than 3,832 cases because an SCI patient might have more than one level of injury. Regarding the severity of the injury, there were more cases of incomplete injuries than complete injuries, and the percentages of complete and incomplete injuries were 17.70% and 82.30%, respectively. This result revealed that the proportion of complete injuries was as follows: 21.8% in the cervical spine, 14.6% in the thoracic spinal region, and 15.0% in the lumbar spine. The distributions of injury severity at the different spinal levels were all statistically significant at *P*<0.05 (**Table 4**).

**Table 4:** Distribution of spine level injuries for SCI patients by the severity of injury.

Level of injury	Severity of injury, n (%)			*P-value*
	Complete	Incomplete	Total	
**Cervical**				<.001
Yes	375 (21.8)	1345 (78.2)	1720 (44.9)	
No	303 (14.3)	1809 (85.7)	2112 (55.1)	
**Thoracic**				<.001
Yes	185 (14.6)	1079 (85.4)	1264 (33.0)	
No	493 (19.2)	2075 (80.8)	2568 (67.0)	
**Lumbar**				0.016
Yes	141 (15.0)	800 (85.0)	941 (24.6)	
No	537 (18.6)	2354 (81.4)	2891 (75.4)	
**Total**	678 (17.7)	3154 (82.3)	3832 (100.0)	

### Associated injuries

As shown in **Table 5**, 73.7% of the SCI patients had associated injuries: spinal fractures were found in 48.6% of the SCI patients, other fractures were reported in 16.0% of the patients, and brain injuries were observed in 9.1% of the patients. Spinal fractures were observed in 23.7% of the patients with complete injuries; 26.3% and 25.4% of the patients with other fractures and brain injuries, respectively, had complete SCIs. Statistically significant differences were noted among all the associated injury groups with regard to the severity of injury (*P*<0.001) (**Table 5**).

**Table 5:** Characteristics of associated injuries in the 3832 patients by the severity of injury.

Associated injury	Severity of injury, n (%)			*P-value*
	Complete	Incomplete	Total	
**Spinal fracture**				<.001
Yes	442 (23.7)	1422 (76.3)	1864 (48.6)	
No	236 (12.0)	1732 (88.0)	1968(51.4)	
**Fracture of other parts**				<.001
Yes	161 (26.3)	452 (73.7)	613 (16.0)	
No	517 (16.1)	2702 (83.9)	3219 (84.0)	
**Brain injury**				<.001
Yes	89 (25.4)	261 (74.6)	350 (9.1)	
No	589 (16.9)	2893 (83.1)	3482 (90.9)	
**Total**	678 (17.7)	3154 (82.3)	3832 (100.0)	

### Clinical complications

In this study, 12.8% of the SCI patients experienced clinical complications. The number of complication cases increased from 13 to 491 cases during the study period. The four main complications were pulmonary infections (37.6%), urinary tract infections (26.3%), bedsores (13.6%), and electrolyte disturbances (10.3%). As shown in **Table 6**, the percentages of patients with complete SCIs who experienced pulmonary infections, urinary tract infections, bedsores, and electrolyte disturbances were 51.4%, 38.0%, 32.8%, and 25.5%, respectively.

**Table 6:** Complications experienced by SCI patients by the severity of injury.*

Complication	Severity of injury, n (%)		Total
	Complete	Incomplete	
Pulmonary infection	95 (51.4)	90 (48.6)	185 (37.6)
Urinary tract infection	49 (38.0)	80 (62.0)	129 (26.3)
Bedsore	22 (32.8)	45 (67.2)	67 (13.6)
Electrolyte disturbance	13 (25.5)	38 (74.5)	51 (10.3)
Deep venous thrombosis	4 (28.6)	10 (71.4)	14 (2.8)
Digestive system disease	4 (28.6)	10 (71.4)	14 (2.8)
Urinary calculus	4 (40.0)	6 (60.0)	10 (2.0)
Spasms	0 (0)	4 (100.0)	4 (0.8)
Autonomic dysreflexia	2 (66.7)	1 (33.3)	3 (0.6)
Cardiovascular diseases	1 (33.3)	2 (66.7)	3 (0.6)
Osteoporosis	0 (0)	1 (100.0)	1 (0.2)
Other	6 (60.0)	4 (40.0)	10 (2.0)
**Total**	200 (40.7)	291 (59.3)	491 (100.0)

* The total number in this table was 491, which referred to the number of patients with complications; the total number (n) of patients in this study was 3832.

### Risk factor analysis of complete SCIs

The risk factors for a complete SCI were investigated by performing a univariate logistic regression analysis. In the model for the analysis, the dependent variable was the severity of the injury, and the independent variables, which included the significant variables from the chi-square tests and the variables considered relevant from modeling expertise (including age group, gender, having a spinal fracture or not, having complications or not, etiology, level of injury, marital status and occupation). The results showed that male gender (OR = 1.33, 95% CI: 1.06–1.67), having a spinal fracture (OR = 2.34, 95% CI: 1.97–2.79), having complications (OR = 3.42, 95% CI: 2.76–4.23), having a cervical injury (OR = 1.69, 95% CI: 1.43–2.00), and having a thoracic injury (OR = 2.25, 95% CI: 1.85–2.73) resulted relatively more often in complete injuries. The unmarried patients were at a higher risk for complete injuries than the married patients (OR = 1.46, 95% CI = 1.15–1.86). In contrast, patients with lumbar injuries (OR = 0.78, 95% CI: 0.64–0.96), retired patients compared with workers, and patients who experienced violence, high falls, traffic accidents, low falls, and non-traumatic injuries compared with patients struck by falling objects had ORs<1 and were therefore more likely to experience incomplete injuries (**Table 7** **&** **Table 8**). Moreover, the multivariate logistic regression model was used to screen the related risk factors for a complete injury. The results revealed that male gender (OR = 1.25, 95% CI: 1.07–1.89), having a spinal fracture (OR = 1.56, 95% CI: 1.35–2.60), having a thoracic injury (OR = 1.23, 95% CI: 1.10–2.00), and having complications (OR = 2.47, 95% CI:1.96–3.13) were the major risk factors for a complete SCI. High falls, traffic accidents, low falls, and non-traumatic injuries, compared with being struck by falling objects, had ORs<1, so they were the protective factors against complete SCIs (**Table 9**).

**Table 7:** Univariate logistic regression analysis of complete injuries.

Variables	*OR* (95%CI)	*P-value*
**Age** a **(y)**		
≤20	0.83 (0.53–1.29)	0.410
21–40	1.08 (0.80–1.47)	0.630
41–60	1.04 (0.77–1.41)	0.780
≥61	1.00 (reference)	-
**Gender**		
Male	**1.33 (1.06–1.67)**	0.013
Female	1.00 (reference)	-
**Spinal fracture**		
Yes	**2.34 (1.97–2.79)**	<.001
No	1.00 (reference)	-
**Complication(s)**		
Yes	**3.42 (2.76–4.23)**	<.001
No	1.00 (reference)	-
**Cervical injury**		
Yes	**1.69 (1.43–2.00)**	<.001
No	1.00 (reference)	-
**Thoracic injury**		
Yes	**2.25 (1.85–2.73)**	<.001
No	1.00 (reference)	-
**Lumbar injury**		
Yes	**0.78 (0.64–0.96)**	0.021
No	1.00 (reference)	-

**NOTE**. *OR* = Odds ratio, *95*% CI = 95% confidence interval.

a A total of 223 patients' medical records were missing age information.

**Table 8:** Univariate logistic regression analysis of complete injuries (continued).

Variables	*OR* (95%CI)	*P-value*
**Etiology**		
Struck by falling objects	1.00 (reference)	-
Violence	0.83 (0.52–1.32)	0.420
High falls	**0.60 (0.43–0.84)**	0.003
Traffic accidents	**0.59 (0.42–0.82)**	0.002
Crushing injuries	0.33 (0.07–1.52)	0.157
Sports-related injuries	0.58 (0.19–1.81)	0.348
Low falls	**0.40 (0.26–0.60)**	<.001
Non-traumatic^a^	**0.06 (0.02–0.18)**	<.001
Other^b^	**0.50 (0.29–0.87)**	0.015
**Marital status**		
Married	1.00 (reference)	-
Unmarried	**1.46 (1.15–1.86)**	0.002
Divorced	3.09 (0.93–10.24)	0.065
Widowed	1.99 (0.41–9.71)	0.396
Unknown	1.16 (0.14–9.74)	0.892
**Occupation**	1.00 (reference)	-
Workers	0.92 (0.74–1.14)	0.459
Peasants	1.25 (0.73–2.12)	0.413
Unemployed	0.80 (0.35–1.83)	0.603
Retired	**0.54 (0.31–0.94)**	0.029
Other occupations^c^	0.71(0.50–1.03)	0.070
Other	0.71 (0.44–1.15)	0.167

**NOTE**. *OR* = Odds ratio, *95*% CI = 95% confidence interval.

a Non-traumatic referred to inflammation, tumors, ossification, degenerative damage, and vascular damage.

b Other included low falls, violence, and sports-related injuries.

c Other occupations included self-employed people, civil servants, students, and enterprise staff.

**Table 9:** Multivariate logistic regression analysis of complete injuries.

Variables	*OR* (95% *CI*)	*P-value*
**Gender**		
Male	**1.25 (1.07–1.89)**	0.034
Female	1.00 (reference)	-
**Spinal fracture**		
Yes	**1.56 (1.35–2.60)**	<.001
No	1.00 (reference)	-
**Thoracic injury**		
Yes	**1.23 (1.10–2.00)**	0.001
No	1.00 (reference)	-
**Complication(s)**		
Yes	**2.47 (1.96–3.13)**	<.001
No	1.00 (reference)	-
**Etiology category**		
Struck by falling objects	1.00 (reference)	-
High falls	**0.46 (0.39–0.71)**	0.009
Traffic accidents	**0.75 (0.48–1.10)**	0.007
Low falls	**0.45 (0.28–0.70)**	<.001
Non-traumatic	**0.23 (0.12–0.76)**	0.006

**NOTE**. The variables incorporated into the logistic regression model included age group, gender, etiology, level of injury, having a spinal fracture or not, having a complication or not, marital status, and occupation.

### Treatment options

Depending on the severity of the injury, it was expected that there would be different treatment options for the SCI patients. In this study, the proportions of patients accepting surgery, rehabilitation therapy, traction, and hyperbaric oxygen therapy were 55.4%, 21.7%, 12.8% and 7.2%, respectively. Among the 678 complete SCI patients, there were 407 patients who received a surgical intervention, 187 patients adopting rehabilitation therapy, 154 patients accepting traction, and 61 patients treated with hyperbaric oxygen therapy. The differences in the number of patients accepting surgery, rehabilitation therapy, and traction between the patients with and without complete injury were statistically significant (*P*<0.05). However, the difference between the two groups in relation to hyperbaric oxygen therapy was not significant (*P*>0.05) (**Table 10**). The prognosis of the SCI patients was categorized as cured, improved, non-cured, and unknown in this study, and the proportions were 41.4%, 51.7%, 3.8%, and 3.1%, respectively. Among the patients who received a surgical intervention, 41.9% were cured and 52.5% improved. The proportion of cured patients was 34.5% among those accepting rehabilitation therapy, 43.6% among those accepting traction therapy, and 22.9% among those adopting hyperbaric oxygen therapy. There were significant differences between the distributions of patients accepting surgery or not, adopting rehabilitation therapy or not, and accepting traction or not by prognosis (*P*<0.05), but there was not a statistically significant difference in prognosis between the group that accepted hyperbaric oxygen therapy and those who did not (*P*>0.05) (**Table 11**).

**Table 10:** Treatment options for SCI patients by the severity of injury.

Treatment	Severity of injury, n (%)			*P-value*
	Complete	Incomplete	Total	
**Surgery**				0.043
Yes	407 (19.1)	1718 (80.8)	2125 (55.4)	
No	271 (15.9)	1436 (84.1)	1707 (44.5)	
**Rehabilitation**				<.001
Yes	187 (22.5)	643 (77.5)	830 (21.7)	
No	491 (16.4)	2511 (83.6)	3002 (78.3)	
**Traction**				<.001
Yes	154 (31.3)	338 (68.7)	491 (12.8)	
No	525 (15.7)	2816 (84.3)	3341 (87.2)	
**HO therapy** *				0.246
Yes	61 (22.2)	214 (77.8)	275 (7.2)	
No	617 (17.3)	2940 (82.7)	3557 (92.8)	
**Total**	678 (17.7)	3154 (82.3)	3832 (100.0)	

* HO therapy = Hyperbaric oxygen therapy.

**Table 11:** Treatment options for SCI patients by prognosis.

Treatment	Prognosis, n (%)					*P-value*
	Cured	Improved	Non-cured	Unknown	Total	
**Surgery**						<.001
Yes	890 (41.9)	1116 (52.5)	74 (3.5)	45 (2.1)	2125 (55.4)	
No	696 (40.8)	864 (50.6)	73 (4.3)	74 (4.3)	1707 (44.5)	
**Rehabilitation**						<.001
Yes	286 (34.5)	496 (59.8)	24 (2.9)	24 (2.9)	830 (21.7)	
No	1300 (43.3)	1484 (49.4)	123 (4.1)	95 (3.2)	3002 (78.3)	
**Traction**						0.069
Yes	214 (43.6)	241 (49.1)	26 (5.3)	10 (2.0)	491 (12.8)	
No	1372 (41.0)	1739 (52.1)	121 (3.6)	109 (3.3)	3341 (87.2)	
**HO therapy** *						0.015
Yes	63 (22.9)	202 (73.5)	8 (2.9)	2 (0.7)	275 (7.2)	
No	1523 (42.8)	1778 (50.0)	139 (3.9)	117 (3.3)	3557 (92.8)	
**Total**	1586 (41.4)	1980 (51.7)	147 (3.8)	119 (3.1)	3832 (100.0)	

* HO therapy = Hyperbaric oxygen therapy.

## Discussion

It is well known that SCI imposes a substantial burden on individuals, their families and society because of the cost of healthcare treatments, rehabilitation and lost productivity. Therefore, the results of this research can support the reasonable allocation of medical resources and the implementation of preventive measures. This study is a retrospective cross-sectional study of the characteristics of the SCI patients in Guangdong, China, from 2003 through 2011. As a retrospective study, it was inevitable that some data might have been lost. The loss of data was minimized by examining all the related medical records to obtain a data set that was as complete as possible. The number of SCIs has displayed a trend of annual growth. The results of the study revealed that the mean age of SCI patients was 42.4±15.3 years, which is in accordance with a 2011 report from Tianjing [14]. This result is more than 5 years older than the mean age reported in North American countries [18]. In this study, the male-to-female ratio of SCI patients was approximately 3.0∶1, while the latest census from Guangdong province in 2011 reported that the male-to-female ratio is 1.1∶1. Most patients fell into the 41- to 60-year-old age group, followed by the 21- to 40-year-old age group. A Canadian literature review reported that most patients fell into the 21- to 40-year-old age group and that the male-to-female ratio of SCI patients was 3–4∶1 [19]. This finding may result from the fact that most women work as homemakers or are engaged in occupations with a low risk of injury. Men are more likely to work in dangerous occupations. This situation is especially true for young men, who perform more dangerous outdoor work. The results revealed that the proportion of married patients is larger than the proportion of patients with other types of marital status, which may be because the largest group of patients was middle-aged, when most Chinese people have married. In this investigation, the common occupational categories were workers (36.2%), peasants (22.8%), and unemployed people (13.9%). This result is similar to a previous report from Tianjing in 2007 that found that the two main categories of SCI patients were unemployed people (48.5%) and peasants (20.1%) [14]. The similarity might be attributed to the fact that peasants and workers were more engaged in dangerous outdoor work. The causes of spinal cord injuries can be seen as the result of differences between countries and between different time periods [4].

Currently, most epidemiological research studies from countries worldwide have focused on traumatic SCIs, and the information about non-traumatic SCIs is limited [20]. In this study, traumatic, non-traumatic (referring to inflammation, tumors, ossification, degenerative damage, and vascular damage) and unknown etiologies were all included to assess the epidemiological characteristics and etiologies. This approach revealed that the main etiologies in Guangdong were traffic accidents (21.6%), being struck by falling objects (19.4%), crushing injuries (15.1%) and high falls (9.7%). Thus, the four main causes of SCIs were all traumatic. These results are in accordance with the 2007 report from Tianjin; in this report, low falls (16.7%) and high falls (35.2%) combined were the leading cause of injury, followed by traffic accidents (36.4%) and being struck by falling objects (5.4%) [14]. The results showed that traffic accidents were the leading cause during the three periods we examined. This result might be due to social modernization, especially the developments in transportation, the increase in population density, and the lack of traffic safety awareness. These factors could lead to quick increases in the traffic accident rate, which has become the major cause of SCI. There have been many recent reports about gender differences in the causes of SCI. Work-related and violence-related injuries happen more often to males [21]. This study revealed that the main etiologies for various occupations were different, which is an important consideration for preventive measures. There were limitations in the assessment of occupational classifications in this study: for example, rural migrant workers might have dual roles as peasants and workers. Furthermore, low fall SCIs occurred mainly in the group aged 60 years or older, and violence largely happened to patients approximately 45 years old [2]. Similarly, there were high rates of traffic accidents, high falls, and being struck by falling objects for the patients between 21 and 60 years old, while low falls were the most common cause of SCI in this study for the participants over 60 years old. Thus, the high-energy injuries, such as traffic accidents and being struck by falling objects, were the major causes of SCI in young adults, whereas low-energy injuries such as low falls mainly occurred in the elderly [22]. As we all know, people are more prone to injuries as they age and their agility declines. Therefore, we should focus on the elderly and high-risk groups to prevent spinal cord injuries. This study also revealed that traffic accidents were the main causes of SCI for both married and unmarried patients, although there were no significant differences in the distribution of marital status by etiology. It was noted in our study that cervical injuries were the most common type of SCI, accounting for 55% to 75% [23], [24]. This result is attributable to the relatively poor mechanical stability of the cervical vertebrae, which makes the cervical spine more vulnerable to trauma than any other area of the vertebral column. The trend in cervical injuries increased in the 1970s; the rate of cervical injuries climbed from 53.3% to 56.5% by 2000 [21]. These results showed that the highest number of SCI patients suffered cervical injuries. With regard to the severity of the injuries, complete spinal cord injuries accounted for approximately two-thirds of the SCIs over the past 30 years, but this figure was reduced to 45% ten years ago in Beijing [12]. This reduction might be attributed to improvements in first aid technology, which allow patients to receive timely and appropriate treatment and avoid aggravating the injury [25]. In the investigation, the percentages of complete and incomplete SCIs were 17.70% and 82.30%, respectively. A Canadian group reported that complete injuries accounted for 35% of SCIs in 2006. The incidence of complete injury was higher in the thoracic segments than in the lumbar and cervical segments [13]. Tianjin reported that 100% of thoracic SCIs resulted in complete injuries, whereas 46.7% and 60% of cervical and lumbar SCIs, respectively, resulted in incomplete injuries. The level of the injury appears to have an effect on the extent of the neurological deficit. The results of the multivariate logistic regression model revealed that the greatest risk factors for a complete injury were male gender, having a spinal fracture, having a thoracic injury, and experiencing complications. A spinal fracture may affect the stability of the spinal mechanics, so patients with spinal fractures are more prone to complications involving the spinal cord or the end of the cauda equine nerve. Thus, the patients with thoracic spinal fractures were more prone to complete injuries. In addition, in the past, complete injuries had longer periods of hospitalization and rehabilitation. Thus, understanding the severity of the injury has guided the use of treatment options for patients with spinal cord injuries of differing severity. Our results demonstrated that the differences in the distribution of treatment options (including surgery, traction therapy, and rehabilitation) by the severity of injury revealed statistically significant differences (*P*<0.05); however, the difference between the two groups in relation to hyperbaric oxygen therapy was not significant (*P*>0.05), and a greater number of patients accepted surgery than other therapies. This result reveals that there is a link between treatment options and the severity of SCI patients' injuries and that surgery is a common therapy for SCI patients, especially for patients with complete injuries. Furthermore, this understanding has helped high-risk patients with complete injuries avoid further injuries that might sever the spinal cord. The treatments used for the patients with and without complete injury were significantly different. Therefore, timely and appropriate hospital treatment (and eventually rehabilitation therapy) should be provided for the high-risk population with complete injuries. A USA study reported that outcomes following surgery immediately after cord injuries were significantly better compared with outcomes for patients who were managed conservatively [26]. It is notable in our study that the patients who had a surgical intervention also had a better prognosis (were cured or improved) than those who did not receive surgery. In addition, two reports on spinal cord injuries revealed that rehabilitation therapy played an important role in promoting recovery from neurological deficits [27], [28]. Although the proportion of patients who were cured with rehabilitation therapy or hyperbaric oxygen therapy was smaller than the group that did not receive an intervention in our study, the results may be due to the smaller number of patients who received rehabilitation or hyperbaric oxygen therapy. SCI is often associated with injuries to other body parts, fractures, and brain injuries [14]. In this study, the percentage of clinical complications was 12.8%, and the four main complications were pulmonary infections (37.6%), urinary tract infections (26.3%), bedsores (13.6%), and electrolyte disturbances (10.3%). In addition, Italy reported that the four main complications of SCI were urinary tract infections, pain, spasms and bedsores [15]. Urinary tract infections and bedsores were common complications, and they were mainly attributed to the poor nursing practices in hospitals. These results emphasize that taking good care of patients is very important for shortening the duration of hospitalization and for improving prognosis.

China is a rapidly developing country, and Guangdong is a region of China that is undergoing significant economic expansion. Compared with other regions, Guangdong has unique characteristics: a larger population, a higher-level economy than most of China, and more engagement with international community. Until now, epidemiology research about spinal cord injuries in Guangdong, China has been very rare. However, this information is needed to implement preventive measures to control the expansion of the SCI population. First, data must be collected and analyzed to help define the problem and to identify possible risk factors in various populations. Preventive strategies should be targeted at persons who are at the greatest risk for injury, such as young male adults who are engaged in dangerous outdoor work. Second, environmental modifications should be strengthened. Roads should be widened and protective barriers installed between the motor road and the sidewalk to prevent traffic accidents [29]. Third, education is needed for persons at risk of injury [30]. Based on demographics and epidemiologic characteristics, different educational programs can be provided for different age/gender/occupational groups. Fourth, strengthening legislation to require the use of protective tools and helmets in high-risk occupations and to punish individuals who violate traffic rules is needed [31]. When necessary, strict traffic rules and related safety laws should be enacted.

## Conclusions

In summary, although there were some shortcomings, the results of the study were similar to another related study about the epidemiology of spinal cord injuries. The mean age at the time of injury was older, and the proportion of males was higher. The main causes were traffic accidents and being struck by falling objects. Workers, peasants and the unemployed were the high-risk occupational groups. The number of SCI patients has increased annually. The statistical analysis revealed that being male, having a spinal fracture, having a thoracic injury, and having complications were the major risk factors for a complete SCI. The treatments were significantly different between the patients with and without complete injuries. All these data indicated that preventive measures should be based on the characteristics of different groups, and public policies aimed at preventing injuries should focus on high-risk populations, such as young males.

## References

[1] O'Connor P (2002) Incidence and patterns of spinal cord injury in Australia. Accid Anal Prev 34: 405–415. 12067103 10.1016/s0001-4575(01)00036-7

[2] Jackson AB, Dijkers M, Devivo MJ, Poczatek RB (2004) A demographic profile of new traumatic spinal cord injury–changes and stablity over 30 years. Arch Phys Med Rehabil 85: 1740–1748. 15520968 10.1016/j.apmr.2004.04.035

[3] Behrman AL, Trimble SA (2012) Outcomes of spinal cord injuries in young children. Dev Med Child Neurol 54: 1078. 23066816 10.1111/j.1469-8749.2012.04440.x

[4] Chiu WT, Lin HC, Lam C, Chu SF, Chiang YH, et al (2010) Review paper: epidemiology of traumatic spinal cord injury: comparisons between developed and developing countries. Asia Pac J Public Health 22: 9–18. 20032030 10.1177/1010539509355470

[5] Albert T, Ravaud JF (2005) Rehabilitation of spinal cord injury in France: a nationwide multicentre study of incidence and regional disparities. Spinal Cord 43: 357–365. 15741980 10.1038/sj.sc.3101717

[6] Dahlberg A, Kotila M, Leppanen P, Kautiainen H, Alaranta H (2005) Prevalence of spinal cord injury in Helsinki. Spinal Cord 43: 47–50. 15520842 10.1038/sj.sc.3101616

[7] Kattail D, Furlan JC, Fehlings MG (2009) Epidemiology and clinical outcomes of acute spine trauma and spinal cord injury: experience from a specialized spine trauma center in Canada in comparison with a large national registry. J Trauma 67: 936–943. 19901651 10.1097/TA.0b013e3181a8b431

[8] Galvin J, Scheinberg AD, New PD (2013) A retrospective case series of pediatric spinal cord injury and disease in Victoria, Australia. Spine (Phila Pa 1976) 10.1097/BRS.0b013e318294e839 23574815

[9] Devivo MJ (2012) Epidemiology of traumatic spinal cord injury: trends and future implications. Spinal Cord 50: 365–372. 22270188 10.1038/sc.2011.178

[10] Chien LC, Wu JC, Chen YC, Liu L, Huang WC, et al (2012) Age, sex, and socio-economic status affect the incidence of pediatric spinal cord injury: an eleven-year national cohort study. PLoS One 7: e39264. 22761749 10.1371/journal.pone.0039264 PMC3382245

[11] Grossman RG, Frankowski RF, Burau KD, Toups EG, Crommett JW, et al (2012) Incidence and severity of acute complications after spinal cord injury. J Neurosurg Spine 17: 119–128. 22985378 10.3171/2012.5.AOSPINE12127

[12] Chun-xia H, Jian-jun L, Hong-jun Z (2007) Epidemiology Characteristics of Spinal Cord Injury in Hospital :1264 Cases Report. Chinese journal of Rehablitation Theory and Practice 13: 1011–1013.

[13] Pickett GE, Campos-Benitez M, Keller JL, Duggal N (2006) Epidemiology of traumatic spinal cord injury in Canada. Spine (Phila Pa 1976) 31: 799–805. 16582854 10.1097/01.brs.0000207258.80129.03

[14] Feng HY, Ning GZ, Feng SQ, Yu TQ, Zhou HX (2011) Epidemiological profile of 239 traumatic spinal cord injury cases over a period of 12 years in Tianjin, China. J Spinal Cord Med 34: 388–394. 21903012 10.1179/2045772311Y.0000000017 PMC3152810

[15] Pagliacci MC, Franceschini M, Di Clemente B, Agosti M, Spizzichino L (2007) A multicentre follow-up of clinical aspects of traumatic spinal cord injury. Spinal Cord 45: 404–410. 17102809 10.1038/sj.sc.3101991

[16] Scivoletto G, Farchi S, Laurenza L, Tamburella F, Molinari M (2013) Impact of multiple injuries on functional and neurological outcomes of patients with spinal cord injury. Scand J Trauma Resusc Emerg Med 21: 42. 23718823 10.1186/1757-7241-21-42 PMC3669625

[17] Ditunno JJ, Young W, Donovan WH, Creasey G (1994) The international standards booklet for neurological and functional classification of spinal cord injury. American Spinal Injury Association. Paraplegia 32: 70–80. 8015848 10.1038/sc.1994.13

[18] Brotherton SS, Krause JS, Nietert PJ (2007) Falls in individuals with incomplete spinal cord injury. Spinal Cord 45: 37–40. 16491105 10.1038/sj.sc.3101909

[19] Ackery A, Tator C, Krassioukov A (2004) A global perspective on spinal cord injury epidemiology. J Neurotrauma 21: 1355–1370. 15672627 10.1089/neu.2004.21.1355

[20] New PW, Simmonds F, Stevermuer T (2011) A population-based study comparing traumatic spinal cord injury and non-traumatic spinal cord injury using a national rehabilitation database. Spinal Cord 49: 397–403. 20603631 10.1038/sc.2010.77

[21] Couris CM, Guilcher SJ, Munce SE, Fung K, Craven BC, et al (2010) Characteristics of adults with incident traumatic spinal cord injury in Ontario, Canada. Spinal Cord 48: 39–44. 19546873 10.1038/sc.2009.77

[22] McDonald JW, Sadowsky C (2002) Spinal-cord injury. Lancet 359: 417–425. 11844532 10.1016/S0140-6736(02)07603-1

[23] Karacan I, Koyuncu H, Pekel O, Sumbuloglu G, Kirnap M, et al (2000) Traumatic spinal cord injuries in Turkey: a nation-wide epidemiological study. Spinal Cord 38: 697–701. 11114778 10.1038/sj.sc.3101064

[24] Dryden DM, Saunders LD, Rowe BH, May LA, Yiannakoulias N, et al (2003) The epidemiology of traumatic spinal cord injury in Alberta, Canada. Can J Neurol Sci 30: 113–121. 12774950 10.1017/s0317167100053373

[25] Spinal cord injury facts and figures at a glance. J Spinal Cord Med 36: 1–2. 23433327 10.1179/1079026813Z.000000000136 PMC3555099

[26] McKinley W, Meade MA, Kirshblum S, Barnard B (2004) Outcomes of early surgical management versus late or no surgical intervention after acute spinal cord injury. Arch Phys Med Rehabil 85: 1818–1825. 15520977 10.1016/j.apmr.2004.04.032

[27] Gittler MS, McKinley WO, Stiens SA, Groah SL, Kirshblum SC (2002) Spinal cord injury medicine. 3. Rehabilitation outcomes. Arch Phys Med Rehabil 83: S65–S71, S90–S98. 11973699 10.1053/apmr.2002.32160

[28] Kirshblum SC, Priebe MM, Ho CH, Scelza WM, Chiodo AE, et al (2007) Spinal cord injury medicine. 3. Rehabilitation phase after acute spinal cord injury. Arch Phys Med Rehabil 88: S62–S70. 17321851 10.1016/j.apmr.2006.12.003

[29] Erdogan MO, Anlas DS, Kosargelir M, Colak S, Ozturk E (2013) Local differences in the epidemiology of traumatic spinal injuries. Ulus Travma Acil Cerrahi Derg 19: 49–52. 23588980 10.5505/tjtes.2013.74501

[30] Richards JS, Hendricks C, Roberts M (1991) Prevention of spinal cord injury: an elementary education approach. J Pediatr Psychol 16: 595–609. 1744808 10.1093/jpepsy/16.5.595

[31] Shin JC, Kim DH, Yu SJ, Yang HE, Yoon SY (2013) Epidemiologic change of patients with spinal cord injury. Ann Rehabil Med 37: 50–56. 23525183 10.5535/arm.2013.37.1.50 PMC3604234

